# *Allium mongolicum* Regel flavonoids promote intestinal health in goats by regulating bile acid metabolism and gut microbiota

**DOI:** 10.1128/msystems.00775-26

**Published:** 2026-07-29

**Authors:** Ruixin Yang, Aihuan Yu, Yaodi Xie, Xiao Zhang, Beibei Guo, Lei Xu, Wenliang Tao, Yue Ma, Yongliang Huang, Yongzhi Cao, Wangjing Liu, Zhaomin Lei

**Affiliations:** 1 College of Animal Science and Technology, Gansu Agricultural University 454422, Lanzhou, Gansu, China; 2 Huan County Animal Husbandry and Veterinary Bureau, Qingyang, China; California State University Stanislaus, Turlock, California, USA; Northwest A&F University, Yangling, China

**Keywords:** Saanen dairy goat, *Allium mongolicum *Regel flavonoids, bile acids, gut microbiota, intestinal immunity

## Abstract

Bile acids (BAs) are processed and modified by intestinal microorganisms into various secondary BAs, which play a crucial role in maintaining intestinal homeostasis. Recent studies indicate that flavonoids influence the gut microbiota–BAs axis to regulate intestinal immunity and improve metabolic health, though the mechanisms remain to be elucidated. The present findings demonstrate that dietary supplementation with *Allium mongolicum* Regel flavonoids significantly reduced the serum concentrations of pro-inflammatory cytokines (TNF-α, IL-1β, and IL-6) and markedly increased the mucosal thickness of the colon in goats. Concomitantly, the expression of tight junction proteins in the colon was upregulated, thereby reinforcing the intestinal physical barrier function. The treatment modulated the balance between T helper 17 cells and T regulatory cells. The concentrations of ursodeoxycholic acid (UDCA), lithocholic acid (LCA), deoxycholic acid (DCA), chenodeoxycholic acid (CDCA), and allo-lithocholic acid (alloLCA) increased in the ileum, while in the colon, the levels of UDCA, LCA, DCA, and CDCA were similarly increased. Dietary supplementation with flavonoids significantly reduced the ACE index of colonic bacteria, without significantly affecting β-diversity of the gut microbiota. The stability and complexity of the ileal and colonic microbiota increased. Signaling pathways such as PI3K-Akt, MAPK, and Wnt were associated with the aforementioned changes and may serve as candidate pathways. In summary, dietary supplementation with *A. mongolicum* Regel flavonoids in goats reduced serum inflammatory cytokines, promoted intestinal health, altered the intestinal bile acid composition (including CDCA, DCA, LCA, UDCA, and alloLCA), and modulated the structure and function of the gut microbiota.

**IMPORTANCE:**

Flavonoids are a class of polyphenolic compounds widely distributed in fruits, vegetables, tea, legumes, and nuts. There exists a complex interplay among bile acids, gut microbiota, and the immune system. Research indicates that flavonoids can ameliorate intestinal health by modulating bile acids metabolism. In this study, dietary supplementation of *Allium mongolicum* Regel flavonoids significantly reduced serum inflammatory factor levels, improved colonic health of Saanen dairy goats, elevated the concentrations of certain intestinal bile acids, and altered the composition and functions of intestinal microbial communities. These findings provide novel and useful insights into the complex interplay among bile acids, gut microbiota, and intestinal immunity while offering a theoretical basis for the utilization of *A. mongolicum* Regel flavonoids as a green feed additive.

## INTRODUCTION

The gastrointestinal tract of ruminants is integral to their overall health, productivity, and welfare (1). The colon is a major site of microbial fermentation and nutrient absorption in ruminants, and it can absorb and utilize more than 10% of carbohydrates through an intricate regulatory network of microorganisms and metabolites (2). In addition, bioactive small molecules metabolically produced or modified by gut microbiota (e.g., short-chain fatty acids, bile acids [BAs], lipids, and amino acid metabolites) can serve as ligands for G protein-coupled receptors and hormone receptors, thereby influencing animal growth and development (3). Meanwhile, the colonic microbiota acts in concert with various components of the colon (such as different types of mucosal epithelial cells, neuromuscular tissues, immune effector cells, etc.) through synergistic interactions to collectively maintain normal colonic function. The functions of the intestine exhibit regional specificity, with nutrient absorption occurring primarily in the small intestine, while the large intestine is closely associated with immunity. In production practice, factors such as weaning stress, pathogenic infection, and high-concentrate diets often induce intestinal damage (4). The onset of such diseases has been linked to disturbances in the host’s gut microbiota and their metabolic outputs. Thus, dietary manipulation of the intestinal microbiota and associated metabolites may represent an effective approach to promote animal health (5).

Bile acids are generally considered to play a key role in the digestion and absorption of fats (6). Recent studies suggest that impaired BA metabolism is a key contributor to the pathogenesis of several gastrointestinal diseases in animals, such as infectious enteritis and post-weaning diarrhea (7). BAs are initially synthesized in the liver, secreted into the small intestine, and subsequently metabolized and modified by the gut microbiota into secondary bile acids (SBAs), which are then reabsorbed via the ileum into the liver (8). While the majority of primary BAs are reabsorbed in the ileum, a portion enters the colon, with a minor fraction ultimately being excreted in feces (9). Primary bile acids (PBAs) undergo further biotransformation in the ileum and colon, a process catalyzed by enzymes derived from the gut microbiota (10). Crucial to gut homeostasis, the high expression of BA receptors in the ileum and colon underpins their significant role in preserving the intestinal barrier and modulating immune responses (11). BAs and their derived metabolites influence T-cell differentiation, notably by modulating the equilibrium between T helper 17 (T_H_17) and regulatory T (T_reg_) cells (12). Zhu et al. (13) found that 24-norursodeoxycholic acid (NorUDCA) ameliorates intestinal inflammation by suppressing the function and differentiation of proinflammatory T_H_17 cells while promoting the formation of functional T_reg_ cells. Song et al. (14) observed that chenodeoxycholic acid (CDCA) supplementation in piglet diets reduced diarrhea incidence, increased the ratio of villus height-to-crypt depth (VH/CD), increased the number of goblet cells and expression of tight junction proteins, and improved intestinal barrier function. Hou et al. (15) reported that supplementing BAs to goats modified the composition of the gut microbiota and metabolites, increased the number of colonic goblet cells, reduced lipopolysaccharide concentration, and ultimately enhanced intestinal mucosal barrier integrity. BAs, as key metabolic intermediates, engage in dynamic interactions with the gut microbiota and are essential for preserving intestinal homeostasis and epithelial barrier integrity.

Flavonoids are a class of polyphenolic compounds widely distributed in fruits, vegetables, tea, legumes, and nuts. Flavonoids are characterized by a chemical structure consisting of two benzene rings linked by an oxygen-containing heterocyclic ring (16). Flavonoids and polyphenols exhibit anti-inflammatory, antioxidant, and antibacterial properties and can be used to prevent and treat chronic inflammatory diseases (17). *Allium mongolicum* is a perennial xerophytic plant belonging to the *Liliaceae* family, known for its rich content of protein, flavonoids, polysaccharides, and other beneficial compounds (18). Our research group established the optimal processing method to extract sufficient flavonoids from *A. mongolicum* Regel, and the optimal supplementation amount in sheep was determined as 2.8 g/day/sheep (19). Flavonoids mainly contain polyphenolic compounds, which are extensively metabolized in the intestine. These metabolites enter hepatic circulation for further processing. The processed metabolites are then transported to target cells, excreted through bile, and hydrolyzed by the intestinal microbiota into glycans, followed by re-entry into enterohepatic circulation. Unabsorbed flavonoid metabolites reach the colon and are degraded and reabsorbed by colon microorganisms (20). Current research indicates that polyphenols can modify the intestinal microbial–BA signaling axis to enhance metabolic health and can thus serve as a novel pharmacological target for regulating BA metabolism (21). Wang et al. (22) demonstrated that the natural polyphenol dihydroquercetin could improve growth performance and restore intestinal damage in piglets infected with *Prevotella copri* by modulating *Prevotella* abundance and BA pool. Liu et al. (23) found that total phenolic extracts of *citrus aurantium* L. had protective and restorative effects on antibiotic-induced intestinal flora dysbiosis, BA metabolism disorders, and intestinal barrier damage in mice. These phenolic extracts maintained BA homeostasis and restored intestinal barrier integrity by regulating the gut–liver axis-related farnesol X receptor (FXR)/fibroblast growth factor 15 pathway and FXR-targeted proteins. These findings confirm the ability of flavonoids to modify the gut microbiota–BAs signaling axis, improve animal intestinal health, and enhance immunity of the animal. Information regarding the effects of flavonoids as green feed additives on the intestinal microbiota, bile acid metabolism, and intestinal health of ruminants remains limited.

Therefore, understanding the effects of *A. mongolicum* Regel flavonoids on the gut microbiota and BA metabolism in goats is crucial for characterizing the role of flavonoids and their impact on goat gut health. We hypothesized that dietary supplementation with *A. mongolicum* Regel flavonoids improves immunity and promotes health in goats by modulating the gut microbiota and BAs. This experiment analyzed serum inflammatory factors, intestinal tissue morphology, immune function, as well as microbial community and bile acid composition in the ileum and colon, to explore the effects of *A. mongolicum* Regel flavonoids on the intestinal health of goats. These findings can provide valuable insights into the relationship between flavonoids and goat health and immunity and offer theoretical references for the development and application of green feed additives.

## MATERIALS AND METHODS

### Experimental materials

The study utilized 12 castrated Saanen dairy goat lambs aged 3 months, with good health and similar weights (16.38 ± 0.74 kg). The animals were randomly divided into two groups via completely randomized design (CRD) and housed in separate cages. Goats in the control (CON) group were fed a basal diet. For goats in the *Allium mongolicum* Regel flavonoid (AMRF) group, 2.8 g of *A. mongolicum* Regel flavonoids per goat per day was supplemented to the basal diet at the optimal dosage determined in a previous study by Liu et al. (18). The flavonoids were extracted from *A. mongolicum* powder using the previously established optimal extraction procedure (19). Fresh *A. mongolicum* was harvested from Minqin County, Gansu Province (36°29′N, 104°16′E). After thorough cleaning, samples were dried to constant weight at 60°C in a constant-temperature drying oven, ground and sieved through a 1 mm mesh, then stored at 4°C. The yield and relative content of flavonoids in dried scallion leaves are shown in Table S1. The extraction process yielded 28% flavonoids from *A. mongolicum* Regel powder. According to the nutritional requirements, the complete diet (Table 1) for dairy goats was formulated using the meat-type sheep and goat feeding standard (NY/T 816-2021) and feed ingredient composition. The experiment included a 15-day pre-trial period, followed by a 124-day formal trial period. Feeding was performed twice daily at 08:00 and 18:00. This study was carried out in Huanxian County, Gansu Province (36°34′N, 107°18′E, altitude 1,500 m), a region with a temperate continental semi-arid climate. The average indoor temperature and relative humidity were 17.0°C and 63%, respectively, throughout the trial, with natural ventilation maintained. The flavonoids were pre-mixed evenly with 200 g of total mixed ration (TMR) pellets to ensure their complete intake by dairy goats; subsequently, the remaining TMR rations were fed. During the trial, the feeding environment for the experimental goats was kept consistent, with a remaining feed of approximately 10% of the feeding amount per meal.

**TABLE 1:** Ingredients and nutrient composition of the experimental diets fed to Saanen dairy goats^*c*^

Items	Fattening stage		
	Phase I (days 1–30)	Phase II (days 31–60)	Phase III (days 61–124)
Ingredients, % of DM			
Corn	25.00	35.00	35.00
Soybean meal	9.00	9.00	17.00
Corn silage	20.00	18.00	11.00
Soybean straw	23.00	17.00	9.00
Oats	13.00	11.00	7.00
Cottonseed meal	9.00	9.00	12.00
Rapeseed meal	–^*d*^	–	8.00
Stone powder	0.75	0.75	0.75
Premix^*a*^	0.25	0.25	0.25
Total	100.00	100.00	100.00
Nutrient levels			
ME, MJ/kg^*b*^	10.13	10.29	11.13
CP	16.28	15.23	16.02
EE	3.00	3.30	3.10
NDF	41.20	39.90	40.02
ADF	25.80	24.80	25.20
Ca	0.79	0.68	0.61
P	0.39	0.38	0.36

^*a*^ Each kilogram of the premix contains the following: 550 kIU of vitamin A, 75 kIU of vitamin D_3_, 6 kIU of vitamin E, 1,600 mg of Cu, 9,400 mg of Fe, 10,400 mg of Mn, 8,000 mg of Zn, 200 mg of I, 60 mg of Se, and 72 mg of Co.

^*b*^ The metabolizable energy (ME) value was calculated according to the standard "Nutrient requirements of meat type sheep and goat" (NY/T816-2021) issued by the Ministry of Agriculture and Rural Affairs of China, while all other indices were determined experimentally.

^*c*^ Abbreviations: ME = metabolizable energy, CP = crude protein, EE = ether extract, NDF = neutral detergent fiber, ADF = acid detergent fiber, Ca = calcium, P = phosphorus.

^*d*^ –, not added (inclusion level = 0).

### Sample collection

After the experiment, all animals were fasted for 12 h. Blood samples (20 mL) were collected from the jugular vein and centrifuged at 1,100 × *g* for 10 min. The upper serum was harvested, aliquoted into 2 mL centrifuge tubes, and stored at −80°C for subsequent analysis. Subsequently, all animals were transported to a commercial abattoir and humanely slaughtered by electrical stunning followed by exsanguination via the jugular vein. After slaughter, all abdominal contents were immediately removed and separately kept. The contents of the ileum and colon were collected, divided into two aliquots, and stored at −80°C for subsequent metagenomic and BA-targeted metabolomic analyses. Colonic spiral loop tissues were collected and fixed in 4% paraformaldehyde for histological morphology, immunohistochemistry, and immunofluorescence analyses.

### Measurement of serum inflammatory factors and complements

Tumor necrosis factor-α (TNF-α), interleukin-1β (IL-1β), interleukin-6 (IL-6), complement 3 (C3), and complement 4 (C4) were measured using commercial kits (Beijing Huaying Biotechnology Research Institute, Beijing, China) according to the manufacturer’s instructions. The levels were determined using a DR-200Bs microplate reader (Wuxi Huawei Delang Instrument Co., Ltd., Wuxi, China).

### Histomorphologic observation

Colonic tissues were fixed, dehydrated, embedded in paraffin, sectioned, sealed, and stained with hematoxylin and eosin. Colon tissue morphology was examined using an optical microscope (Nikon Eclipse E100, Japan), and images were captured using an imaging system (Nikon DS-U3, Japan). CaseViewer v2.4.0 software was used to measure the thickness of the colonic muscle layer and mucosal layer. For statistical analysis, each sample from each group was stained, and 10 fields of view were randomly selected.

### Immunohistochemical and immunofluorescence analyses

Immunohistochemical staining of colon tight junction proteins was performed as follows. Tissue sections were dewaxed, subjected to antigen retrieval, and serum-mounted, followed by the application of rabbit ZO-1 and Occludin primary antibodies (Servicebio, Hubei, China). Subsequently, species-specific secondary antibodies (Servicebio) corresponding to the primary antibodies were applied. DAB was utilized for color development, followed by hematoxylin for counterstaining of cell nuclei. Colonic tight junction proteins were observed using a microscope (Nikon Eclipse E100, Japan). Immunofluorescence techniques were used to analyze T_H_17 and T_reg_ cell–specific antibodies and identify these cell types in colon tissues. Following paraffin embedding, antigen retrieval, and serum blocking, the colon tissue sections were incubated overnight at 4°C with primary antibodies (Servicebio) against IL-17 and FOXP3 raised in rabbit. Secondary antibodies were then applied, and the cell nuclei were counterstained with DAPI (Servicebio). After quenching the spontaneous fluorescence of the tissue, the sections were sealed with an anti-fluorescence quenching sealing agent. The sections were observed with a standing fluorescence microscope (Nikon Eclipse C1, Japan) and scanned with a scanner (3DHISTECH Pannoramic MIDI, Hungary). The positive tissue percentage was calculated using ImageJ version 1.54p software. Each sample from each group was stained, and 10 fields were randomly selected for statistical analysis of the percentage of positive tissue.

### Targeted metabolome analysis of BAs in intestinal contents

A 50 mg aliquot of intestinal content sample was taken and combined with 5 mL of extraction solution (methanol:water:formic acid; 15:4:1 with 0.5% butylated hydroxytoluene). The mixture was vortex-mixed for 1 min, followed by 30 min of ultrasonic treatment at 12,000 rpm for 10 min. During the adsorption step, the supernatant was passed through the SPE solid-phase extraction column at a flow rate below 1 mL/min. The rinsing step was carried out with 3 mL of water and 3 mL of 10% methanol aqueous solution, respectively. The analytes were then eluted with 1 mL of methanol. The eluent was evaporated to dryness and then reconstituted in 0.2 mL of methanol (containing 50 ng/mL cholic acid_D4 as the internal standard). After 1 min of vortex-mixing and centrifugation at 12,000 rpm for 10 min, the supernatant was analyzed by LC-MS/MS. The analysis was performed using a Waters Acquity UPLC system interfaced with an AB SCIEX 5500 QQQ-MS mass spectrometer. Two columns, Acquity UPLC BEH C18 (1.7 μm, 2.1 mm × 100 mm) and Acquity UPLC HSS T3 (1.8 μm, 2.1 mm × 100 mm), were used for chromatographic separation. The column temperature was set at 35°C with a flow rate of 0.30 mL/min. The mobile phase was composed of 10 mM ammonium formate aqueous solution (phase A) and methanol (phase B). The mass spectrometric conditions were as follows: ion source, ESI; ion spray voltage, 4,500 V; ion source temperature, 450°C; ion source gases, 55 arb each for Ion Source Gas1 and Ion Source Gas2. Semi-quantification was performed using the internal standard method, and the points were integrated using MultiQuant software. This method involves adding a known quantity of a pure substance (internal standard) to the sample, determining the target component’s content using the mass ratio between the analyte and the standard, the chromatographic peak area ratio, and the relative correction factor. Chromatographic analysis was performed on the prepared solution containing the analyte and internal standard. The resulting peak responses were utilized to quantify the target component.

### Metagenomic analysis of the gut microbiota

A 0.2 g aliquot of ileum and colon contents was subjected to total genomic DNA extraction using the TIAN Microbe Magnetic Enviro-DNA Kit 4 (Tiangen, Beijing, China). DNA concentration and purity were determined using a Synergy HTX and a NanoDrop 2000 spectrophotometer, respectively. The integrity of the DNA was assessed by electrophoresis on a 1% agarose gel. The DNA sample was sheared into approximately 350-bp fragments by using a Covaris M220 ultrasonicator (Gene Company Ltd., China) to prepare for paired-end sequencing. The sequencing library was then constructed using the NEXTFLEX Rapid DNA-Seq Kit (Bioo Scientific, Austin, TX, USA). DNA libraries were sequenced in the paired-end mode on a NovaSeq X Plus system (Illumina, San Diego, CA, USA) at Majorbio Bio-Pharm Technology Co., Ltd. (Shanghai, China) by utilizing the NovaSeq X Series 25B Reagent Kit (300 Cycle) (Illumina, San Diego, CA, USA) in accordance with the standard protocol (www.illumina.com). The raw data were subjected to trimming and filtering (24). The remaining reads were aligned to the GCF_001704415.2 goat reference genome using BWA (Version 0.7.17) (25). Reads that mapped to the genome, along with their mate pairs, were subsequently removed. Subsequently, the reads were assembled using MEGAHIT (Version 1.1.2) (26). Contigs shorter than 300 bp were discarded, and the remaining sequences were used as the final assembly. Prodigal (Version 2.6.3) was utilized to identify open reading frames in the assembled contigs, and only those exceeding 100 bp were retained (27). The gene catalog was deduplicated using CD-HIT (Version 4.7) with thresholds of 90% sequence similarity and 90% coverage (28). The gene abundance in each sample was quantified using SOAPaligner (Version soap2.21release) with a 95% sequence identity threshold (29). Taxonomic annotation of non-redundant genes was performed using DIAMOND (Version 2.0.13) against the NCBI NR database, with a stringent e-value threshold of 1e-5 for alignment (30). Functional characterization of the non-redundant gene set was performed using the Kyoto Encyclopedia of Genes and Genomes (KEGG) database. Differential analysis was performed using the Kruskal-Wallis test across taxonomic classification, functional categories, and individual genes. The enrichment significance was initially evaluated using the unadjusted *P* value, and pathways with *P* < 0.05 were considered as candidate pathways for further interpretation. To account for multiple testing, false discovery rate (FDR) correction was additionally applied, and the FDR-adjusted *q* values were provided in the supplemental table.

### Statistical analysis

Statistical analysis of serum inflammatory factors, complement, colon mucosal layer thickness, and percentage of positive tissues in immunohistochemical and immunofluorescence analyses was subjected to Student’s *t*-test using SPSS 27.0 software. Results are expressed as mean and standard error. Prior to statistical analysis, the data were tested for normality and homogeneity of variance. Statistical significance was set at *P* < 0.05, with tendencies noted at 0.05 ≤ *P* < 0.10. The Wilcoxon rank-sum test was conducted to analyze the composition of BAs and microbial community parameters (including ACE index and Shannon index). Differences were considered statistically significant when FDR < 0.05. The principal coordinate analysis (PCoA) method is employed to examine the similarities and differences among bacterial communities. Potential biomarkers were screened by using linear discriminant analysis (LDA), with an LDA value greater than 2.5 as the criterion. To further validate the key differentially abundant taxa identified by LEfSe, DESeq2 analysis was performed using the criteria of ∣log_2_ FC∣ >1 and FDR < 0.05. Correlation analysis was performed among microorganisms and between microorganisms and BAs, and Spearman’s correlation coefficient was calculated, with ∣*r*∣ >0.6 and *P* < 0.05 indicating a significant correlation.

## RESULTS

### Effects of *A. mongolicum* Regel flavonoids on serum inflammatory factors and complement in dairy goats

Dietary supplementation with *A. mongolicum* Regel flavonoids significantly (*P* < 0.05) decreased the serum levels of the inflammatory factors TNF-α, IL-1β, and IL-6 in goats (Table 2). It indicates that *A. mongolicum* Regel flavonoids can alleviate systemic inflammatory responses and improve the health status of goats.

**TABLE 2:** Effects of *A. mongolicum* Regel flavonoids on serum inflammatory factors and complement in dairy goats^*a*^

Items	CON	AMRF	SEM	*P*-value
TNF-α (pg/mL)	76.47	40.70	6.74	0.002
IL-1β (pg/mL)	22.59	17.81	1.02	0.017
IL-6 (pg/mL)	126.65	101.86	5.18	0.008
C3 (g/L)	0.43	0.29	0.07	0.349
C4 (g/L)	0.10	0.08	0.02	0.544

^*a*^ CON = control group, AMRF = *A. mongolicum* Regel flavonoid group, TNF-α = tumor necrosis factor-α, IL = interleukin, C = complement.

### Effect of *A. mongolicum* Regel flavonoids on the histomorphology of the colon of dairy goats

**Fig 1:**
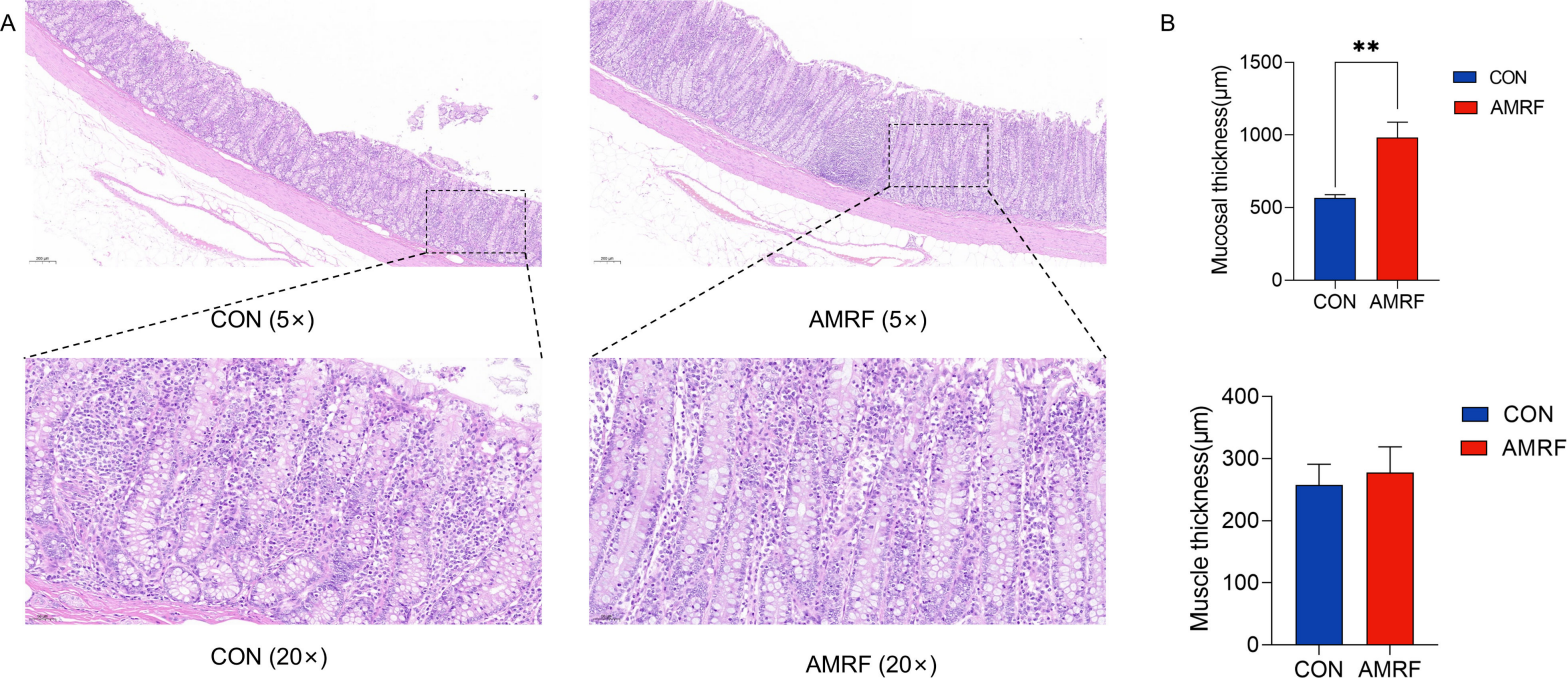
Effects of *A. mongolicum* Regel flavonoids on colonic tissue morphology in dairy goats. (**A**) Histological sections of goat colon from the CON and AMRF groups (5× and 20× fields of view). (**B**) Statistical analysis of colon mucosal and muscle layer thickness. Statistical significance is reported as **P < 0.01. Abbreviations: CON, basal diet group; AMRF, *Allium mongolicum* Regel flavonoid group.

Histomorphological analysis of the colon revealed reduced mucosal lymphocytes in the AMRF group, accompanied by a more organized arrangement of the mucosal epithelium (Fig. 1A). Furthermore, the AMRF group exhibited significantly increased mucosal layer thickness (*P* < 0.01), while muscle layer thickness remained unchanged (Fig. 1B).

### Effects of *A. mongolicum* Regel flavonoids on colon tight junction proteins and T_H_17/T_reg_ cells in goat colon

**Fig 2:**
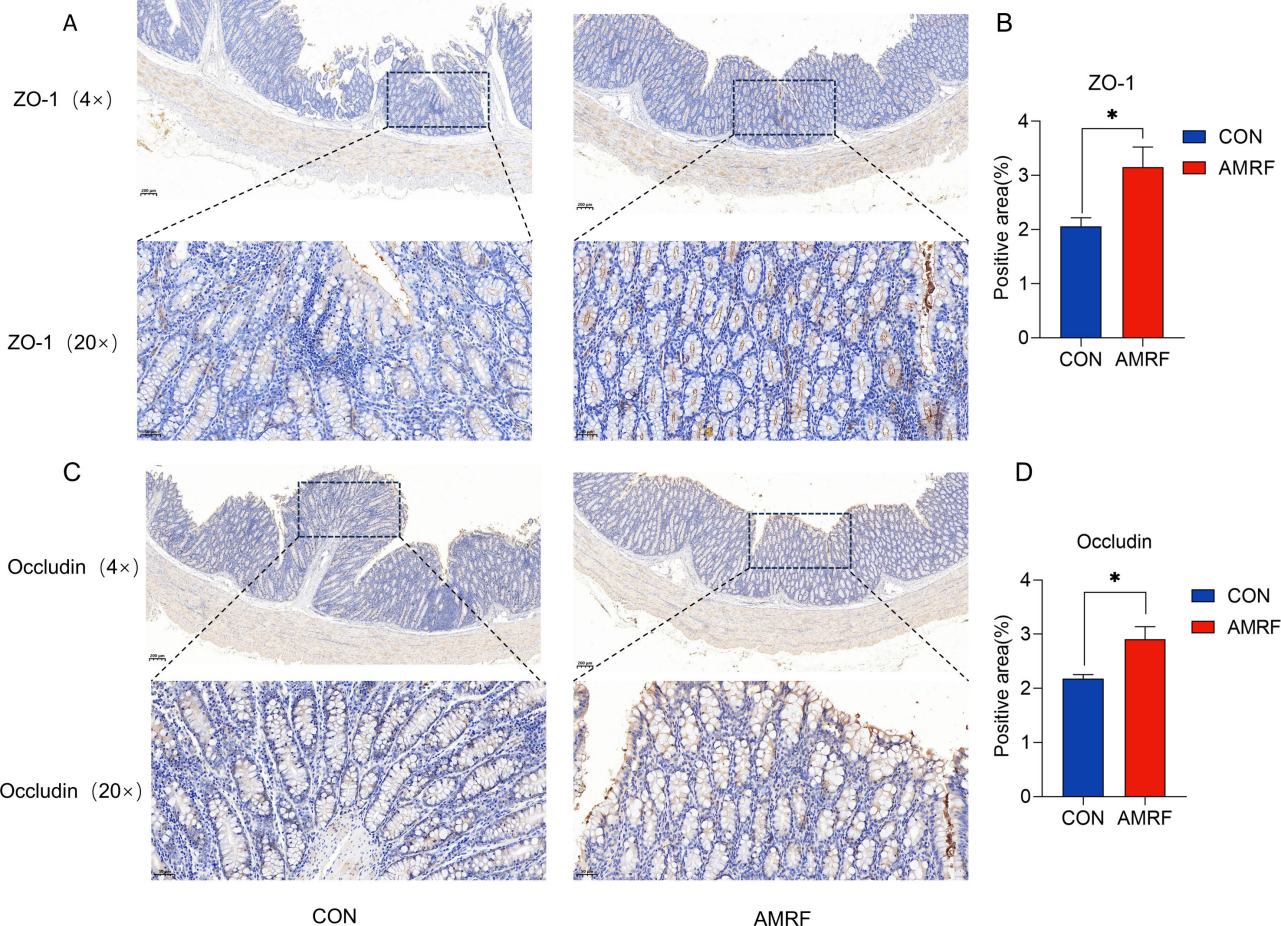
Effects of *A. mongolicum* Regel flavonoids on colonic tight junction proteins in dairy goats. Detection of colon tight junction proteins ZO-1 (**A**) and occludin (**C**) by immunohistochemical analysis. Percentage of ZO-1-positive tissues (**B**) and occludin-positive tissues (**D**). Significance is reported as *P < 0.05, **P < 0.01. Abbreviations: CON, basal diet group; AMRF, *Allium mongolicum* Regel flavonoids group.

Immunohistochemical analysis showed the distribution of colon tight junction proteins ZO-1 (Fig. 2A) and occludin (Fig. 2C). Differential analysis of ZO-1- and occludin-positive tissues (Fig. 2B and D, respectively) indicated that dietary supplementation with the flavonoids significantly enhanced the expression and distribution of tight junction proteins in the colon of goats.

To examine the impact of flavonoids on intestinal immunity in goats, we conducted immunofluorescence staining to detect T_H_17 and T_reg_ cells in goat colon tissues (Fig. S1A). The AMRF group showed significantly reduced positive tissue percentage of T_H_17 cell marker IL-17, compared to the CON group (*P* < 0.01). Additionally, the positive tissue area of the T_reg_ cell marker FOXP3 increased significantly in the AMRF group (*P* < 0.05) relative to that in the CON group (Fig. S1B).

### Effect of *A. mongolicum* Regel flavonoids on BAs metabolism in the ileum and colon of goats

**Fig 3:**
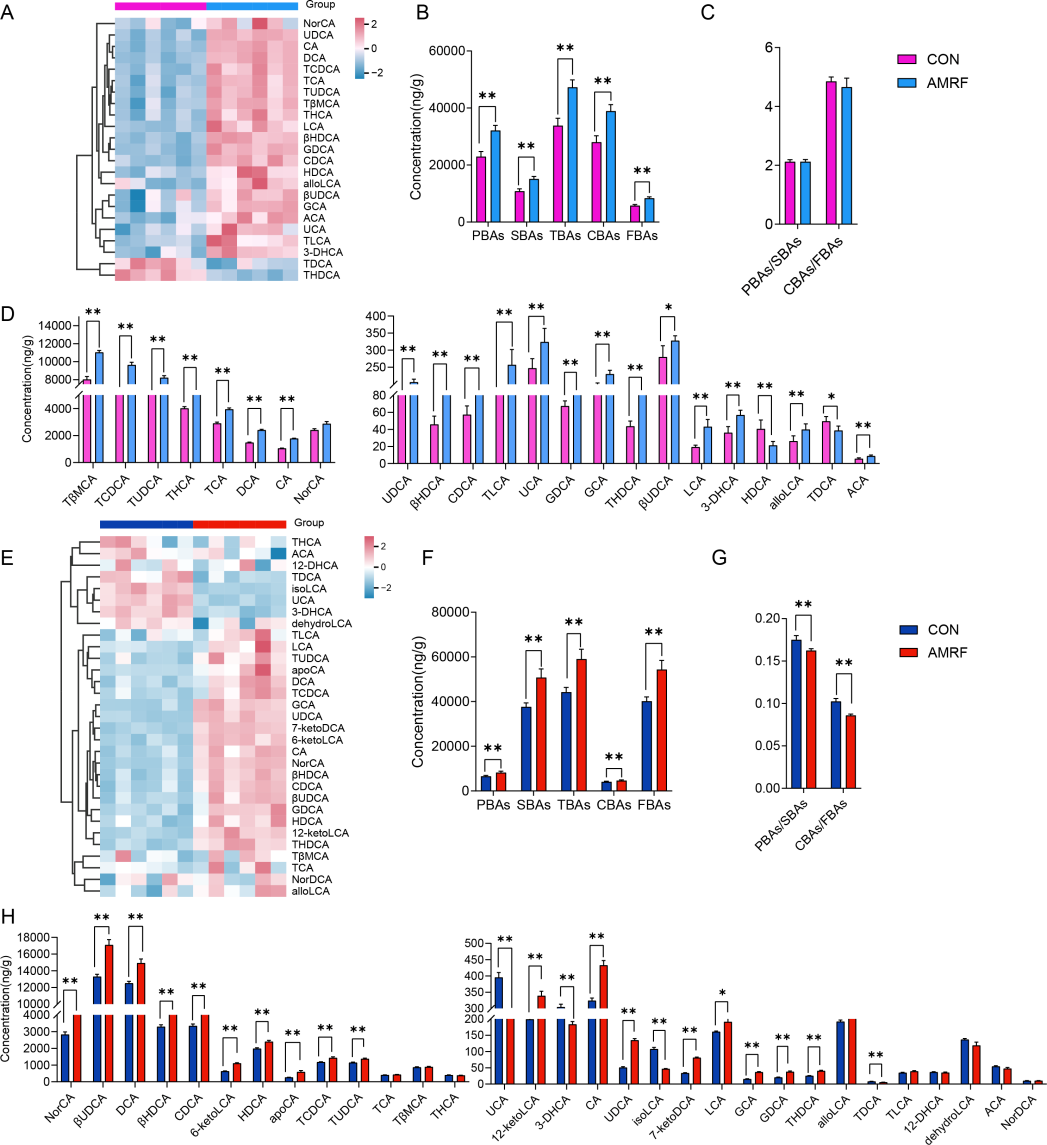
Effects of *Allium mongolicum* Regel flavonoids on intestinal bile acid content in dairy goats. (**A**) Heatmap of BA contents in the ileum. (**B**) Bar chart of classified bile acids in the ileum. (**C**) Bar graph of ratios between primary/secondary and conjugated/free bile acids in the ileum. (**D**) Bar chart of bile acid contents in the ileum. (**E**) Heatmap of BA contents in the colon. (**F**) Bar chart of classified bile acids in the colon. (**G**) Bar graph of ratios between primary/secondary and conjugated/free bile acids in the colon. (**H**) Bar chart of bile acid contents in the colon. Significance is reported as * FDR < 0.05, ** FDR < 0.01. PBAs: primary bile acids, SBAs: secondary bile acids, TBAs: total bile acids, CBAs: conjugated bile acids, FBAs: free bile acids, TβMCA: tauro-β-muricholic acid, TCDCA: taurochenodeoxycholic acid, TUDCA: tauroursodeoxycholic acid, THCA: tetrahydrocannabinolic acid, TCA: taurocholic acid, DCA: deoxycholic acid, CA: cholic acid, NorCA: norcholic acid, UDCA: ursodeoxycholic acid, βHDCA: β-hyodeoxycholic acid, CDCA: chenodeoxycholic acid, TLCA: taurolithocholic acid, UCA: ursocholic acid, GDCA: glycoursodeoxycholic acid, GCA: glycocholic acid, THDCA: taurohyodeoxycholic acid, βUDCA: β-ursodeoxycholic acid, LCA: lithocholic acid, 3-DHCA: 3-dehydrocholic acid, HDCA: hyodeoxycholic acid, alloLCA: allolithocholic acid, TDCA: taurodeoxycholic acid, ACA: aminocaproic acid, 6-ketoLCA: 6-ketolithocholic acid, apoCA: apocholic acid, 12-ketoLCA: 12-ketolithocholic acid, isoLCA: isolithocholic acid, 7-ketoDCA: 7-ketodeoxycholic acid, 12-DHCA: 12-dehydrocholic acid, dehydroLCA: dehydro lithocholic acid, ACA: allocholic acid, NorDCA: nordeoxycholic acid.

Based on results from multiomics technologies such as metabolomics and metagenomics, BAs serve as key signaling molecules within the chemical network that mediates crosstalk between the gut microbiota and the host immune system (31). We investigated the changes in BA metabolism in the ileum and colon. Unsupervised principal component analysis (PCA) and supervised orthogonal partial least squares-hierarchical discriminant analysis (OPLS-DA), which identified changes in BA content in the ileum and colon, revealed significant differences between the two groups (Fig. S2). Twenty-three BAs were identified in the ileum (Fig. 3A). Compared with the CON group, the contents of PBAs, SBAs, conjugated bile acids (CBAs), free bile acids (FBAs), and total bile acids (TBAs) were significantly increased (FDR < 0.05) in the AMRF group. No significant differences were observed in the ratios of primary to secondary bile acids and conjugated to free bile acids (Fig. 3B and C). Compared to the CON group, the AMRF group demonstrated significantly higher concentrations of 20 BAs (FDR < 0.05), including tauro-β-muricholic acid (TβMCA), taurochenodeoxycholic acid (TCDCA), tauroursodeoxycholic acid (TUDCA), tetrahydrocannabinolic acid (THCA), taurocholic acid (TCA), deoxycholic acid (DCA), cholic acid (CA), ursodeoxycholic acid (UDCA), β-hyodeoxycholic acid (βHDCA), CDCA, taurolithocholic acid (TLCA), ursocholic acid (UCA), glycoursodeoxycholic acid (GDCA), glycocholic acid (GCA), taurohyodeoxycholic acid (THDCA), β-ursodeoxycholic acid (βUDCA), lithocholic acid (LCA), 3-dehydrocholic acid (3-DHCA), allolithocholic acid (alloLCA), and aminocaproic acid (ACA) (Fig. 3C; Table S2). Two BAs, namely hyodeoxycholic acid (HDCA) and taurodeoxycholic acid (TDCA), showed decreased levels in the ileum (Fig. 3D; Table S2).

A total of 31 BAs were identified in the colon (Fig. 3E). Of these, 22 BAs showed significant changes in their content (FDR < 0.05). Compared with the CON group, the levels of PBAs, SBAs, CBAs, FBAs, and TBAs were markedly elevated in the AMRF group, while the ratios of primary to secondary bile acids and conjugated to free bile acids decreased significantly (FDR < 0.05) (Fig. 3F and G). The concentrations of NorCA, βUDCA, DCA, βHDCA, CDCA, 6-ketolithocholic acid (6-ketoLCA), HDCA, apocholic acid (apoCA), TCDCA, TUDCA, 12-ketolithocholic acid (12-ketoLCA), CA, UDCA, 7-ketodeoxycholic acid (7-ketoDCA), LCA, GCA, GDCA, and THDCA were significantly higher in the AMRF group than in the CON group. The contents of four BAs were decreased, namely UCA, 3-DHCA, isolithocholic acid (isoLCA), and TDCA (Fig. 3H; Table S3).

### Effect of *A. mongolicum* Regel flavonoids on the intestinal microbiome of dairy goats

**Fig 4:**
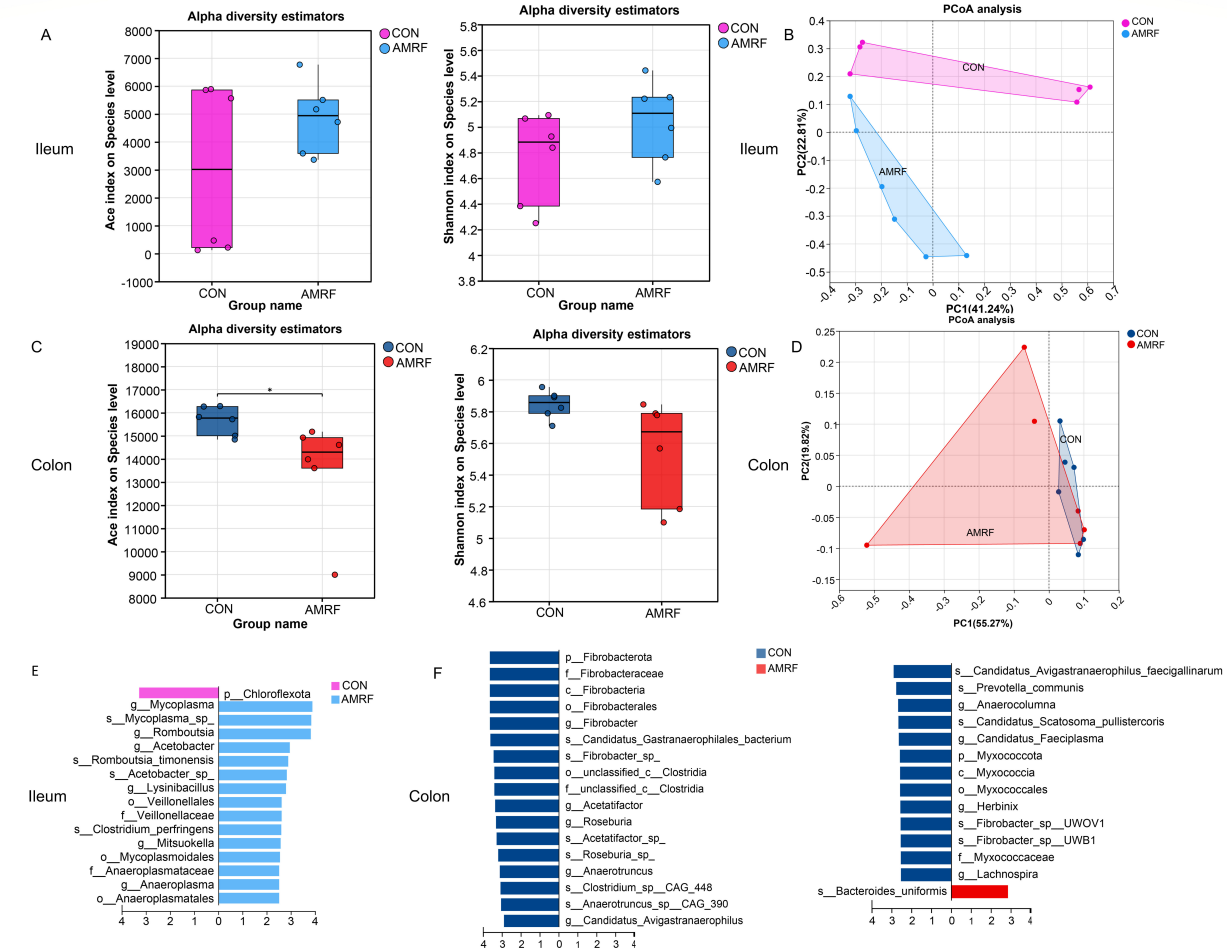
Effects of *Allium mongolicum* Regel flavonoid on intestinal microbial diversity and biomarkers in dairy goats. (**A**) ACE index and Shannon index for bacterial communities in the ileum. (**B**) PCoA plot for bacterial communities in the ileum. (**C**) ACE index and Shannon index for bacterial communities in the colon. (**D**) PCoA plot for bacterial communities in the colon. Biomarkers of microbial communities in the ileum (**E**) and colon (**F**). Significance is reported as * FDR < 0.05. Abbreviations: CON, basal diet group; AMRF, *Allium mongolicum* Regel flavonoids group.

Neither alpha- nor beta-diversity of the ileal microbiota differed significantly between the two groups (Fig. 4A and B). In contrast to the CON group, the AMRF group exhibited a significantly lower ACE index in the colon (FDR < 0.05), whereas the beta-diversity remained comparable between the two groups (Fig. 4C and D). Bacterial composition analysis revealed 135 phyla, 2,723 genera, and 10,883 species in the ileum. The top 20 phyla, genera, and species with relative bacterial abundance in the ileum are presented in Fig. S3A through C. Flavonoid supplementation altered the distribution of dominant phyla in the goat ileum. The second dominant phylum in the ileum changed from Actinomycetota to Bacteroidota (Fig. S3A). A total of 159 phyla, 3,900 genera, and 23,083 species were identified in the goat colon. The top 20 phyla, genera, and species with relative bacterial abundance in the colon are shown in Fig. S3D through F. The dominant bacterial phyla in the colon were Bacillota and Bacteroidota (Fig. S3D). The predominant genera in the colon were *Bacteroides*, *Ruminococcus*, and *Alistipes* (Fig. S3E). Potential biomarkers in the ileum and colon were identified using LEfSe analysis (LDA score >2.5). In the ileum, Chloroflexota emerged as a unique phylum in the CON group, with a significant increase in its abundance. The bacterial species specific to the ileum of the AMRF group included *Mycoplasma*_sp, *Romboutasia_timonensis*, *Acetobacter*_sp, and *Clostridium_perfringens* (Fig. 4E). In the colon of the AMRF group, *Bacteroides_uniformis* was identified as the unique strain, with a significant increase in its relative abundance. The microbial species unique to the CON group were *Candidatus_Gastranaerophilales_bacterium*, *Fibrobacter*_sp, *Acetatifactor*_sp, *Roseburia*_sp, *Clostridium*_sp_CAG:448, *Anaerotruncus*_sp_CAG_390, *Candidatus_Avigastranaerophilus_faecigallinarum*, *Prevotella_communis*, *Candidatus_Scatosoma_pullistercoris*, *Fibrobacter*_sp_UWOV1, and *Fibrobacter*_sp_UWB1 (Fig. 4F). Using the DESeq 2 method (∣Log_2_ FC ∣ >1, FDR < 0.05) for further validation of the aforementioned key bacteria, s__*Mycoplasma* sp., s__*Romboutsia timonensis*, and s__*Acetobacter* sp. were ultimately identified as biomarkers in the ileum of the AMRF group (Fig. S4A). In the colon, s__*Bacteroides uniformis* was identified as a biomarker in the CON group, while s__*Candidatus Avigastranaerophilus faecigallinarum* and s__*Prevotella communis* as core biomarkers in the AMRF group (Fig. S4B).

**Fig 5:**
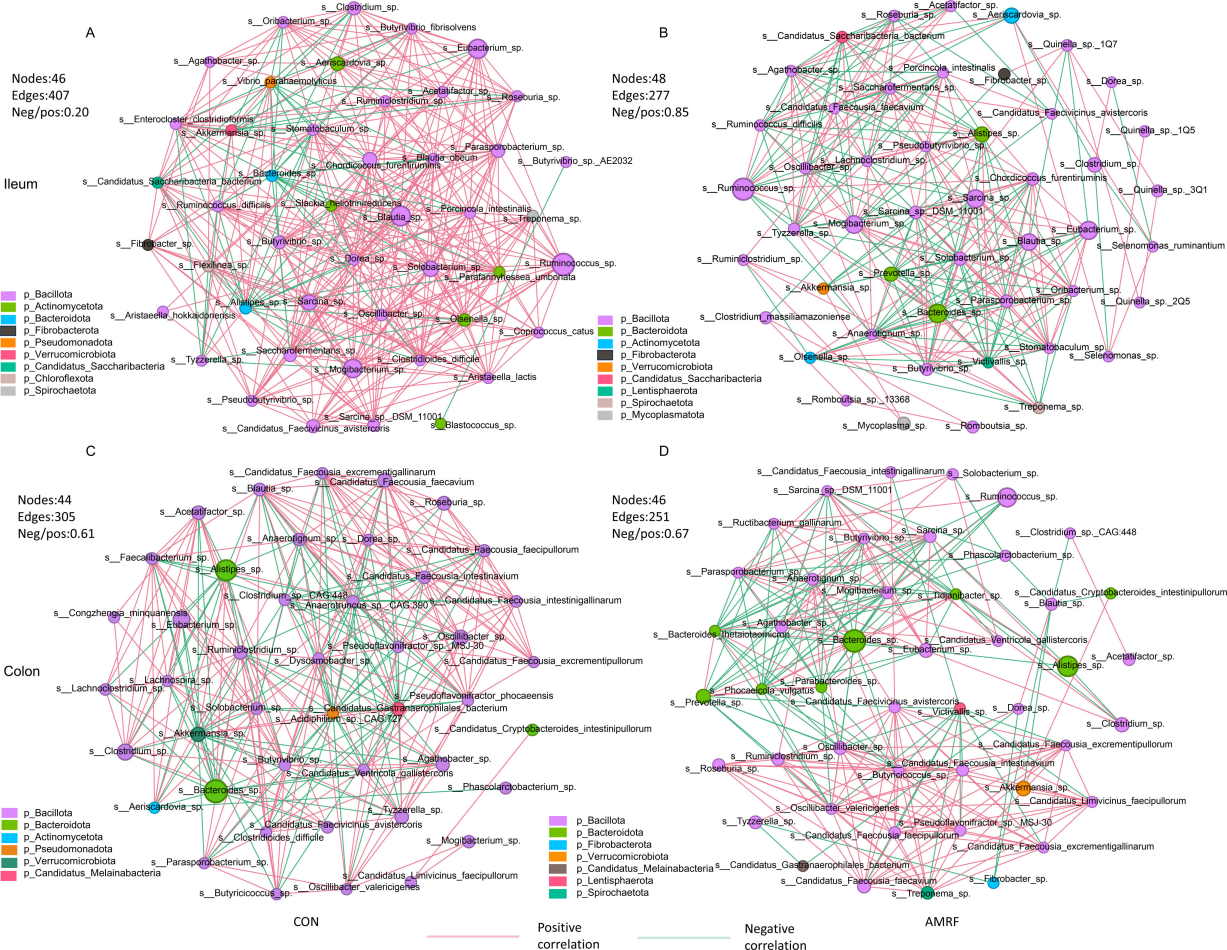
Co-occurrence network analysis of the top 50 most abundant bacterial taxa in the gastrointestinal tract of dairy goats. Ileum of the CON and AMRF groups (**A and B**), respectively. Colon of the CON and AMRF groups (**C and D**), respectively. Nodes represent species, different colors represent different phyla, the red line indicates positive correlation, and the green line indicates negative correlation (Spearman’s correlation coefficient, |*r*| > 0.6, *P* < 0.05).

The co-occurrence networks and network roles of the top 50 bacteria in terms of relative abundance in the ileum and colon were analyzed (Fig. 5). We used the number of nodes to express network complexity. Higher values indicated greater complexity of the network. The ratio of negative correlation to positive correlation (neg/pos) was used to reflect the stability of the network, with larger values indicating a more stable network. The complexity and stability of ileal and colonic microbial co-occurrence networks increased following supplementation with *A. mongolicum* Regel flavonoids. Core microbial communities were identified based on the following three central coefficient values: degree centrality, closeness centrality, and betweenness centrality of all nodes in the network. Higher values indicated greater node importance within the network. In the ileum of the CON group, *s_Chordicoccus_furentiruminis*, *s_Blautia_obeum*, *s_Olsenella*_sp, *s_Porcincola_intestinalis*, and *s_Stomatobaculum*_sp constituted the core microbiota (Fig. 5A). In the ileum of the AMRF group, *s_Sarcina*_sp, *s_Sarcina*_sp._DSM_11001, *s_Bacteroides*_sp, *s_Solobacterium*_sp, *s_Anaerotignum_*sp, *s_Chordicoccus_furentiruminis*, and *s_Alistipes*_sp represented the core microbiota (Fig. 5B). In the colon of the CON group, *s_Candidatus_Gastranaerophilales_bacterium*, *s_Acidiphilium*_sp._CAG:727, *s_Dysosmobacter*_sp, *s_Anaerotruncus*_sp._CAG:390, *s_Akkermansia*_sp, *s_Ruminiclostridium*_sp, *s_Solobacterium*_sp, and *s_Butyrivibrio*_sp comprised the core microbiota (Fig. 5C). In the colon of the AMRF group, *s_Oscillibacter*_sp, *s_Butyricicoccus*_sp, *s_Oscillibacter_valericigenes*, *s_Bacteroides*_sp, *s_Bacteroides_thetaiotaomicron*, *s_Parabacteroides*_sp, and *s_Phocaeicola_vulgatus* formed the core microbiota (Fig. 5D). The core intestinal microbiota composition was altered following dietary supplementation with *A. mongolicum* Regel flavonoids.

**Fig 6:**
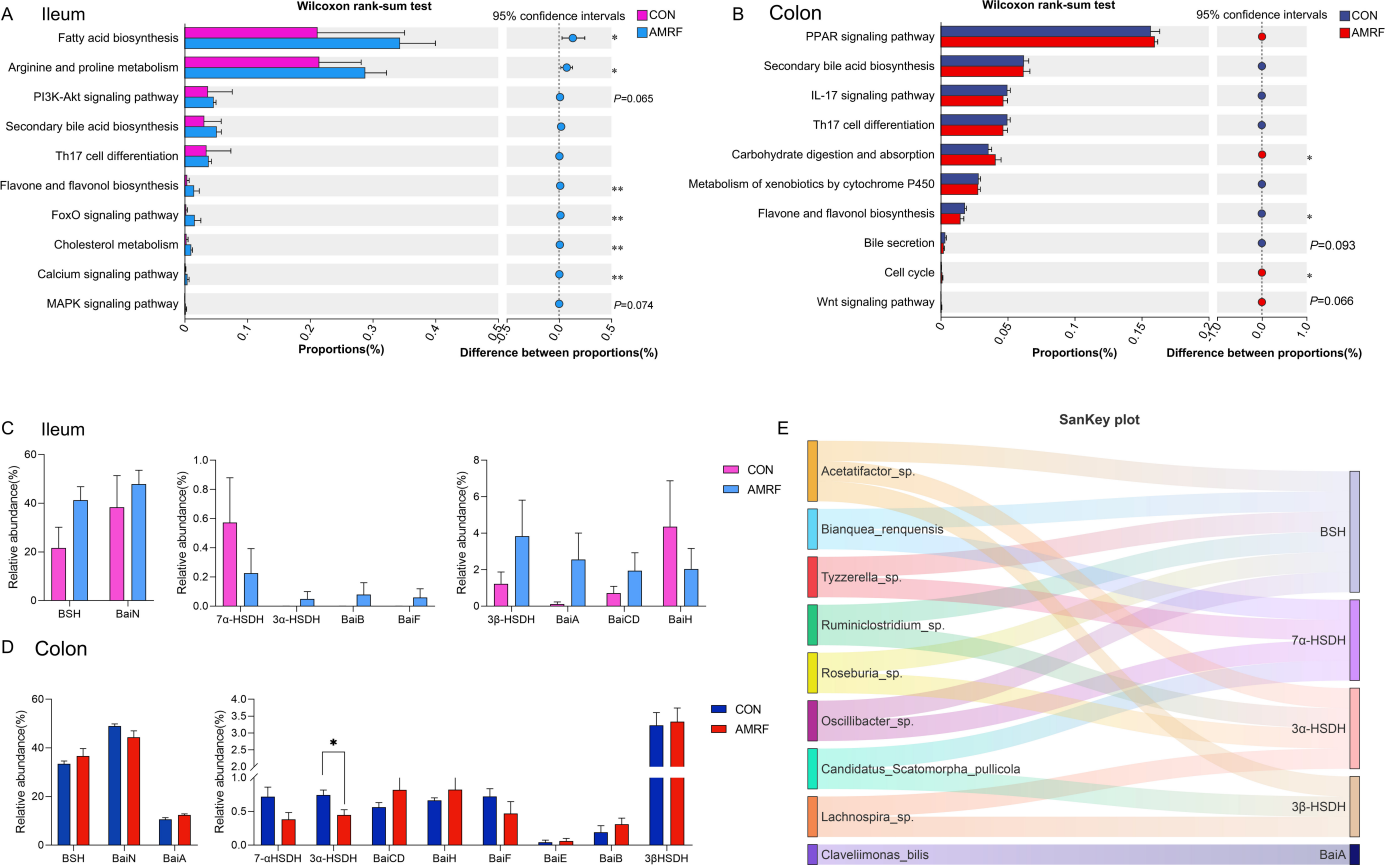
Effects of *Allium mongolicum* Regel flavonoids on intestinal bacterial functions and bile acid-metabolizing enzymes in dairy goats. Results of KEGG pathway enrichment analysis of microbial communities in the ileum (**A**) and colon (**B**). Key enzymes involved in the biosynthesis of secondary BAs in the ileum (**C**) and colon (**D**). Sankey diagram (**E**) shows bacteria with genes encoding key enzymes catalyzing the biosynthesis of secondary BAs. Significance is reported as *P < 0.05, **P < 0.01. Abbreviations: CON: basal diet group, AMRF: *A. mongolicum* Regel flavonoids group. BSH: bile salt hydrolase, BaiN: 3-dehydro-bile acid delta (4, 6)-reductase, 7α-HSDH: 7α-hydroxysteroid dehydrogenase, 3α-HSDH: 3α-hydroxycholanate dehydrogenase, BaiB: bile acid-coenzyme A ligase, BaiF: bile acid CoA-transferase, 3β-HSDH: 3β-hydroxycholanate 3-dehydrogenase, BaiA: 3α-hydroxycholanate dehydrogenase, BaiCD: 3-oxocholoyl-CoA 4-desaturase, BaiH: 7β-Hydroxy-3-oxochol-24-oyl-CoA 4-desaturase, BaiE: 3-oxo-5β-cholanate 4,5-dehydrogenase.

The KEGG pathways involved in the ileal microbiota included fatty acid biosynthesis, arginine and proline metabolism, the PI3K-Akt signaling pathway, secondary bile acid biosynthesis, flavone and flavonol biosynthesis, the FoxO signaling pathway, cholesterol metabolism, the calcium signaling pathway, and the MAPK signaling pathway (Fig. 6A; Table S4). The KEGG pathways involved in the colonic microbiota included the PPAR signaling pathway, secondary bile acid biosynthesis, the IL-17 signaling pathway, Th17 cell differentiation, carbohydrate digestion and absorption, flavone and flavonol biosynthesis, bile secretion, the cell cycle, and the Wnt signaling pathway (Fig. 6B; Table S5). Although these pathways did not reach statistical significance after FDR correction, some of them (e.g., fatty acid biosynthesis, flavone and flavonol biosynthesis, MAPK signaling pathway, and Wnt signaling pathway) remained nominally significant (*P* < 0.05) or showed a trend toward significance (0.05 ≤ *P* < 0.1) and were therefore considered candidate pathways for further discussion. In the ileum, the expression levels of key enzymes related to BAs metabolism were increased, except for those of 7α-hydroxysteroid dehydrogenase and 7β-hydroxy-3-oxochol-24-oyl-CoA 4-desaturase; however, the difference was not significant (*P* > 0.05) (Fig. 6C). In the colon, the expression level of 3α-hydroxycholanate dehydrogenase in the AMRF group was significantly reduced as compared to that in the CON group (*P* < 0.05) (Fig. 6D). The results showed that dietary supplementation with *A. mongolicum* Regel flavonoids altered the expression levels of BAs metabolism-related enzymes in the intestine. Novel bacteria expressing BAs metabolism-related enzymes were identified, including *Bianquea_requensis*, *Candidatus_Scatomorpha_pullicola*, and *Claveliimonas_bilis* (Fig. 6E).

**Fig 7:**
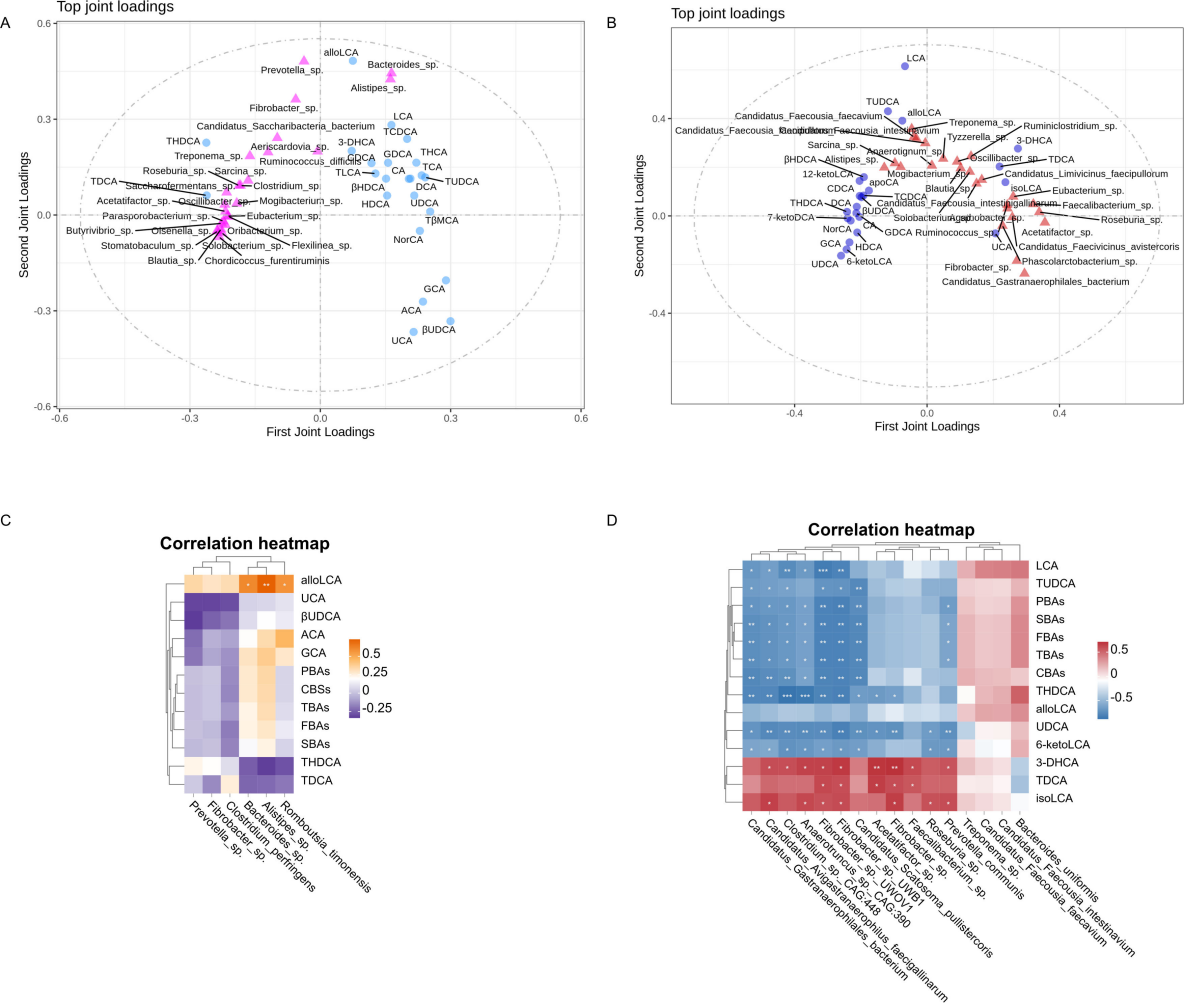
Correlation analysis between gut microbiota and BAs in dairy goats. (**A**) O2PLS analysis of BAs and the ileal microbiota. (**B**) O2PLS analysis of BAs and the colonic microbiota. (**C**) Correlation analysis between BAs and the ileal microbiota. (**D**) Correlation analysis between BAs and the colonic microbiota (Spearman’s correlation coefficient, |*r*| > 0.6, *P* < 0.05). Significance is reported as *P < 0.05, **P < 0.01, *** *P* < 0.001.

The relationship between metabolomic and metagenome data was examined by two-way orthogonal partial least squares (O2PLS) analysis (Fig. 7A and B). Based on the O2PLS results and the potential biomarker selected through microbial LEfSe screening, correlation patterns between microorganisms and BAs were investigated. In the ileum, alloLCA demonstrated a significant positive correlation with *Romboutsia_timonensis*, *Alistipes*_sp, and *Bacteroides*_sp (Fig. 7C). LCA was negatively correlated with *Candidatus_Gastranaerophilales_bacterium*, *Candidatus_Avigastranaerophilus_faecigallinarum*, and *Anaerotruncus_*sp.CAG:390 in the colon (Fig. 7D). LCA showed strong negative correlations with *Clostridium*_sp_CAG:448, *Fibrobacter*_sp._UWB1, and *Fibrobacter*_sp._UWOV1. IsoLCA displayed positive correlations with *Candidatus_Avigastranaerophilus_faecigallinarum*, *Anaerotruncus*_sp_CAG:390, *Fibrobacter_*sp._UWB1, *Fibrobacter_*sp_UWOV1, *Fibrobacter_*sp, *Prevotella_communis*, and *Roseburia_*sp. UDCA demonstrated negative correlations with *Candidatus_Gastranaerophilales_bacterium*, *Acetatifactor_*sp, and *Roseburia_*sp and showed strong negative correlations with *Candidatus_Scatosoma_pullistercoris*, *Candidatus_Avigastranaerophilus_faecigallinarum*, *Anaerotruncus*_sp.CAG:390, *Clostridium_*sp_CAG:448, *Fibrobacter_*sp._UWB1, *Fibrobacter_*sp, and *Prevotella_communis*. TUDCA exhibited negative correlations with *Candidatus_Gastranaerophilales_bacterium*, *Candidatus_Avigastranaerophilus_faecigallinarum*, *Clostridium_*sp_CAG:448, *Fibrobacter_*sp._UWB1, and *Fibrobacter_*sp._UWOV1 and exhibited a strong negative correlation with *Candidatus_Scatosoma_pullistercoris*. Furthermore, in the colon, various bile acid categories (PBAs, SBAs, FBAs, CBAs, and TBAs) were significantly negatively correlated with *Fibrobacter* sp. UWB1, *Fibrobacter*_sp._UWOV1, and *Candidatus Scatosoma pullistercoris*.

## DISCUSSION

Intestinal microorganisms and their metabolites are crucial for maintaining the host’s intestinal immune homeostasis. Common microbial derivatives include BAs, volatile fatty acids, and tryptophan (32). BAs act as endogenous ligands for cell membranes and receptors, exerting pronounced effects on bile acid homeostasis, intestinal barrier integrity, and immune cell tolerance in the gut (33). This study investigated the effects of *A. mongolicum* Regel flavonoids on bile acid metabolism and intestinal immunity in goats.

In our previous study, it was confirmed that dietary supplementation with *A. mongolicum* Regel flavonoids can enhance body weight as well as the average daily gain (ADG) of dairy goats (34). This result indicated the potential of these flavonoids as an effective dietary supplement to promote the growth and development of animals. Mu et al. (35) reported that incorporating 11–33 mg/kg *A. mongolicum* Regel flavonoids into the diet enhanced the ADG and daily feed intake of sheep and reduced the feed-to-gain ratio. The primary reason for this improvement is that flavonoids increase the serum levels of hormones such as growth hormone and insulin-like growth factor-1. Hao et al. (36) reported that supplementing diets with 3 g/kg of sea buckthorn flavonoids significantly improved ADG, dry matter intake, and dry matter digestibility in Hu sheep. Sea buckthorn flavonoids primarily consist of isorhamnetin, quercetin, and kaempferol. Flavonoids from *A. mongolicum* Regel also contain quercetin and other active compounds. Various bioactive substances (isorhamnetin, cyanidin 3-O-rutinoside, chrysoeriol 7-O-hexoside, and tricin 7-O-hexoside) in flavonoids may contribute to antioxidant effects through rumen microbiota modulation (37). Furthermore, research indicates that flavonoids function as plant estrogens that promote animal growth, regulate immune function through neuroendocrine pathways, enhance animal disease resistance, and significantly impact animal production and immunity (35). The present study also found that *A. mongolicum* Regel flavonoids reduced the serum levels of TNF-α, IL-1β, and IL-6. Inflammatory biomarkers serve as indicators of homeostasis in animals, providing valuable insights into their physiological status. In the presence of endotoxin, the activation of signaling pathways such as NF-κB leads to the secretion of pro-inflammatory cytokines, and this increase in inflammatory factors can lead to tissue damage via monocytes and macrophages (38). Studies have shown that flavonoids reduce the incidence of disease by negatively regulating the inflammatory factor IL-1β. Furthermore, compounds such as quercetin, kaempferol, and isorhamnetin, which are present in flavonoids, can enhance animal immunity and reduce inflammation (39). On the other hand, isorhamnetin can inhibit the production of pro-inflammatory cytokines by suppressing the TLR4/NF-κB signaling pathway (40). Similarly, Wang et al. (22) reported that supplementation with dihydroquercetin in piglets, and Zhao et al. (41) found that dietary inclusion of citrus flavonoid extracts in dairy cows significantly reduced the levels of inflammatory factors in the animals. These results indicate that *A. mongolicum* Regel flavonoids enhance goat health and growth by regulating immune responses and reducing pro-inflammatory cytokine levels.

The colon is a major site of microbial colonization and fermentation in ruminants and plays a vital role in energy metabolism, immune regulation, and overall physiological development. However, it is susceptible to influences from dietary and environmental factors, which can lead to conditions such as diarrhea and weight loss in animals (42). It is therefore essential to employ interventions that enhance intestinal immunity, thereby promoting gut health. The ability of flavonoids to modulate the gut microbiota, exert anti-inflammatory effects, and provide antioxidant activity may serve as an important mechanism for preventing the onset of colon inflammation and thereby promoting colon health (16). In the present study, histomorphological analysis of dairy goat colons revealed that dietary supplementation with flavonoids significantly increased colonic mucosal thickness and improved lymphocyte arrangement. Previous research has demonstrated that adding flavonoids from *A. mongolicum* Regel significantly enhanced the relative expression of *beta-defensin*-1 gene in the jejunum and ileum, and *beta-defensin-*2 gene in the jejunum and duodenum of mutton sheep, thereby improving the immune function of these intestinal regions (43). Furthermore, mutton sheep fed with *A. mongolicum* Regel flavonoids exhibited significantly elevated serum levels of interleukin-1β (IL-1β), IL-2, IL-6, and interferon-γ (44). These findings indicate that *A. mongolicum* Regel flavonoids promote intestinal health in dairy goats. Quercetin exerts beneficial effects by regulating the composition of the intestinal microflora, including enhancing intestinal barrier function, reducing inflammation, and decreasing intestinal permeability (22). The expression of colonic tight junction proteins (ZO-1 and occludin) was significantly upregulated in the present study. Since the flavonoids from *A. mongolicum* also contain quercetin, this suggests that these flavonoids may similarly possess the potential benefit of enhancing the intestinal physical barrier function. T_H_17 cells and their secreted cytokines are widely known to drive pro-inflammatory responses and contribute to the pathogenesis of intestinal inflammation (45). Flavonoid intake modulates T-cell differentiation and affects the subsequent activation and infiltration of proinflammatory immune cells in the colon (16). Consumption of flavonoid-rich aronia berry suppressed the differentiation of T_H_1 and T_H_17 cells and attenuated inflammation in a mouse model of dextran sulfate sodium (DSS)-induced colitis (46). T_reg_ cells perform immunomodulatory functions by suppressing immune responses and promoting immune tolerance. T_H_17 and T_reg_ cells demonstrate mutual transformation capabilities under specific conditions, and this dynamic balance is a critical feature of immune system plasticity (47). Immunofluorescence staining of T_H_17 and T_reg_ cell markers in colon tissue was conducted to investigate the effect of *A. mongolicum* Regel flavonoids on T_H_17/T_reg_ cell balance. The results revealed that dietary supplementation with these flavonoids decreased the number of T_H_17 cells in the colon tissue of goats. The IL-17-positive area was significantly reduced, while the FOXP3-positive area was significantly increased after flavonoid supplementation. This finding indicates that *A. mongolicum* Regel flavonoids potentially promote the polarization of naive CD4^+^ T cells into T_reg_ cells. UDCA inhibits T_H_17 cell differentiation and promotes T_reg_ cell proliferation by regulating the expression of IL-17/PPAR signaling pathway-related genes (8). Song et al. (48) reported that mice treated with CDCA, LCA, and UDCA displayed abundant ROR γ t^+^ pT_reg_ cells in the colon. In the present study, the content of BAs in goat intestine was altered following flavonoid supplementation, with a significant increase in the levels of CDCA, UDCA, and LCA. This finding suggests that *A. mongolicum* Regel flavonoids may influence naive CD4^+^ T-cell differentiation through BAs content regulation.

Flavonoids from *A. mongolicum* Regel, primarily comprising polyphenols, represent a significant non-nutritive plant-derived compound. As reported previously, dietary polyphenols can alter the composition of the intestinal microbial community, BA pool, and BA signaling (21). Analysis of BAs composition in intestinal contents showed that the BAs composition of the ileum and colon was significantly altered following dietary supplementation with flavonoids from *A. mongolicum* Regel. The majority of BAs showed a significant increase, with exceptions noted in the ileum (decreased content of HDCA and TDCA) and colon (decreased content of UCA, 3-DHCA, isoLCA, and TDCA). These differential results demonstrate that flavonoids from *A. mongolicum* Regel influence BAs metabolism and enterohepatic circulation in dairy goats. In addition, the contents of PBAs, SBAs, TBAs, CBAs, and FBAs were significantly elevated in the ileum and colon, while the ratios of PBAs/SBAs and CBAs/FBAs decreased markedly in the colon. Previous studies have shown that flavonoids may regulate transcription factors and nuclear receptors involved in bile acid metabolism, thereby upregulating CYP7A1 expression and promoting bile acid synthesis (49). This may explain why the levels of various bile acids were significantly increased in the AMRF group in the present study. The transformation from PBAs to SBAs and CBAs to FBAs relies on microbial participation (6). These ratio variations may reflect enhanced intestinal microbial activity in goats after supplementation with *A. mongolicum* Regel flavonoids. UDCA, an endogenous hydrophilic BA, demonstrates therapeutic potential in treating cholestasis diseases, stimulating bile flow, and ameliorating DSS-induced colitis in mice (50). The current study demonstrated a significant elevation in UDCA levels within the ileum. LCA and DCA show anti-inflammatory effects in IBD patients, and previous studies have demonstrated that these compounds can mitigate ulcerative colitis by activating Takeda G protein-coupled receptor 5 in the intestine (51). Both LCA and DCA levels increased significantly in the ileum. On the other hand, studies have also shown that increased concentrations of LCA and DCA lead to higher relative abundances of *Lactobacillus* and *Bifidobacterium* at the genus level, potentially contributing to human diseases (52). Furthermore, elevated DCA levels affect innate immunity, and DCA dose-dependently promotes Th17 cell differentiation and the production of pro-inflammatory cytokines *in vitro* (53). In the present study, observations of colonic tissue morphology and immunohistochemistry revealed no intestinal damage or inflammation. In contrast, serum levels of inflammatory cytokines were reduced in the AMRF group, indicating that the increased concentrations of LCA and DCA did not adversely affect the health of goats. In future studies, further in-depth investigation is warranted to explore the impacts of changes in LCA and DCA concentrations. CDCA shows protective effects against lipopolysaccharide-induced intestinal epithelial barrier dysfunction in IPEC-J2 cells and mouse models (54). Additional research indicates that dietary CDCA supplementation enhances the VH/CD, increases the number of mucopolysaccharide-secreting goblet cells to form a protective mucus layer that maintains intestinal epithelial barrier integrity (14). These findings suggest that appropriate intestinal CDCA concentrations maintain barrier function. The present study observed a significant increase in both ileal and colonic CDCA contents following supplementation with flavonoids from *A. mongolicum* Regel. These alterations in BA content suggest potential improvements in animal immunity and intestinal health following dietary supplementation with *A. mongolicum* Regel flavonoids. Similar findings were reported by Wang et al. (22), who demonstrated that dihydroquercetin (flavonoids) ameliorates intestinal injury by modulating the intestinal flora and BAs pool composition. Zhao et al. (41) demonstrated that citrus flavonoid extract reduces systemic inflammatory response and botulinum toxin levels in dairy cows while improving immune metabolism. The regulatory effects of *A. mongolicum* Regel flavonoids on the intestinal BAs pool probably originate from the characteristic poor bioavailability of polyphenols, with metabolic benefits apparently mediated by regulation of the intestinal microflora and polyphenol-derived microbial metabolites (21). These flavonoids effectively modulate the intestinal microflora–BA signaling axis, thereby promoting metabolic health.

Flavonoids and their metabolites can alter the intestinal microflora by suppressing pathogen growth while enhancing the growth of beneficial microflora, particularly *Bifidobacterium* and *Lactobacillus*. Intestinal microorganisms contribute to intestinal health through multiple mechanisms: reducing endotoxin production, facilitating primary-to-secondary BAs conversion, maintaining immune homeostasis, and enhancing nutrient absorption (16). Our findings revealed that following the administration of *A. mongolicum* Regel flavonoids, the second dominant phylum in the ileum shifted from Actinomycetota to Bacteroidota. Bacteroidota represents essential symbiotic bacteria in the gut, which are crucial for maintaining microbiota balance and host health. Extensive research has demonstrated complex interactions between intestinal bacteria and BAs, which subsequently influence intestinal immunity (55–57). Based on these observations, we conducted further analysis of bacteria in the goat ileum and colon. The analysis of the ileal microbiota indicated that *A. mongolicum* Regel flavonoids did not significantly alter the alpha-diversity of ileal bacterial communities. PCoA demonstrated differences in bacterial communities between the groups, with partial separation. The AMRF group exhibited a significant decrease in the ACE index of the colonic bacterial communities, although the PCoA plot showed no significant separation. These findings suggest that *A. mongolicum* Regel flavonoids influence the composition of the intestinal bacteria. The AMRF group showed a higher abundance of certain ileal bacterial species, including *Mycoplasma*, *Romboutsia_timonensis,* and *Acetobacter*_sp. In the colon, *Bacteroides_uniformis* abundance increased significantly in the AMRF group, while *Candidatus_Gastranaerophilales_bacterium*, *Fibrobacter*_sp, *Candidatus _Avigastranaerophilus_faecigllinarum,* and *Prevotella_communis* showed a significant increase in abundance in the CON group. On the other hand, *Clostridium perfringens* was also identified as a potential biomarker in the ileum of the AMRF group by LEfSe analysis. The significant enrichment of *Mycoplasma sp*. and *Clostridium perfringens* may pose a potential risk to goat health. However, based on the growth performance and serum levels of inflammatory cytokines and complement factors during the experimental period, no adverse effects on goat health were observed. Further studies are warranted to investigate the impact of *A. mongolicum* Regel flavonoids on ileal health in goats. BAs also function as antimicrobial agents that directly affect the composition of the gut microbiota. Conversely, alterations in the intestinal microbial community significantly influence BAs metabolism (10). Administration of *A. mongolicum* Regel flavonoids has been shown to exert regulatory effects on the BA pool in goats. Subsequent analysis of secondary BAs biosynthesis pathways revealed changes in key enzymes involved in secondary BAs metabolism, and bacteria expressing BAs metabolism-related enzymes were identified. These results suggest that *A. mongolicum* Regel flavonoids may selectively modulate intestinal microbial communities in goats, promoting the enrichment of specific bacterial groups. Co-occurrence network analysis of bacterial communities further demonstrated that *A. mongolicum* Regel flavonoids enhanced the stability and complexity of the networks formed by goat intestinal bacterial communities.

KEGG pathway enrichment analysis revealed that, following dietary supplementation with *A. mongolicum* Regel flavonoids in goats, the major metabolic pathways in which the intestinal microbiota participated included fatty acid biosynthesis, arginine and proline metabolism, and carbohydrate digestion and absorption. Dietary flavonoids frequently interact with various compounds, including carbohydrates, fats, proteins, and acids (58). Previous research has demonstrated that flavonoids affect multiple molecular targets by regulating the AMPK and Wnt signaling pathways and blocking the cell cycle at specific stages of adipocyte development. While the majority of studies have demonstrated the anti-adipogenic properties of flavonoids, some specific compounds within this class have been shown to promote adipogenic differentiation in mesenchymal stem cells or preadipocytes (59). Our findings indicate that *A. mongolicum* Regel flavonoids altered the metabolism of fatty acids and other nutrients in the goat intestine. This modification may contribute significantly to the growth-promoting effects of *A. mongolicum* Regel flavonoids in goats. The supplementation process significantly altered the biosynthesis of flavanones and flavonols in the goat intestine, suggesting that exogenous *A. mongolicum* Regel flavonoids may influence flavonoid metabolism. Notably, the biosynthetic pathways of flavonoids and flavonols showed contrasting trends in the ileum and colon, potentially relating to flavonoid absorption and utilization. As indicated previously, tea flavonoid absorption in the small intestine is relatively low, with absorption and processing occurring in the large intestine through interactions with the colonic microflora (58). In the AMRF group, the PI3K-Akt, MAPK, and Wnt signaling pathways were still considered candidate signaling pathways. Polyphenols possess anti-inflammatory, antibacterial, antioxidant, and neuroprotective properties. Polyphenols counteract cytotoxicity and apoptosis while regulating innate and adaptive immunity through their immunomodulatory characteristics (60). Most chronic inflammatory conditions are associated with chronic inflammatory diseases, and inhibiting chronic inflammation could be an alternative treatment approach (61). Therefore, the anti-inflammatory properties of polyphenols may significantly facilitate chronic disease prevention. As noted above, the beneficial effects of polyphenols may be associated with enhanced signal transduction of the PI3K-Akt pathway. The trend toward a higher average relative abundance of this pathway in the AMRF group compared with the CON group may represent a mechanism through which polyphenols exert their beneficial effects (62). Although several KEGG pathways did not remain significant after FDR correction, they may still be biologically meaningful. In particular, fatty acid biosynthesis, cholesterol metabolism, PPAR signaling, and secondary bile acid biosynthesis are closely related to intestinal lipid utilization and host–microbiota metabolic interactions. In addition, inflammatory and signaling pathways such as IL-17, Th17 differentiation, PI3K-Akt, MAPK, and Wnt may reflect changes in immune regulation and epithelial homeostasis. In summary, polyphenolic compounds may exert potentially beneficial effects on intestinal immune status and gut health by modulating the gut microbiota and bile acid metabolism; however, these signaling pathways require further validation in subsequent studies.

Correlation analysis between BAs and intestinal bacteria provides novel insights into their relationship. For instance, alloLCA showed positive correlation with *Romboutsia_timonensis*, *Bacteroides*_sp, and *Alistipes*_sp in the ileum. *Romboutsia* exhibits multiple metabolic functions, including carbohydrate metabolism and amino acid fermentation, ultimately contributing to host growth promotion and health maintenance (63). *Bacteroides* represents the most prevalent and abundant mammalian gut bacteria. *Bacteroides* alleviates colitis by regulating BA metabolism and inhibiting IL-17 signaling pathway activation and T_H_17 cell differentiation (64). A significant negative correlation was observed between *Clostridium*_sp_ACG:448 and LCA in the colon. *Clostridium* expresses bile salt hydrolase and plays a crucial role in BA metabolism (65). In summary, *A. mongolicum* Regel flavonoids may exert beneficial effects on goat growth by promoting the metabolism of carbohydrates and amino acids in the gut and by enhancing intestinal health. Limited research exists regarding the effects of *A. mongolicum* Regel flavonoids on ruminant gut health, which restricts comparative analysis. Future research should further investigate the effects of CDCA, DCA, LCA, and UDCA on intestinal immune function in ruminants. On the other hand, although the ileum and colon are key sites for bile acid metabolism, and the colon also plays an important role in flavonoid metabolism, goats, as ruminants, may be influenced by rumen microorganisms after consuming *A. mongolicum* Regel flavonoids. The absence of rumen microbial or metabolite-related data in the present study may be a limitation. Therefore, future studies should further integrate rumen-related data to elucidate the underlying mechanisms.

### Conclusions

**Fig 8:**
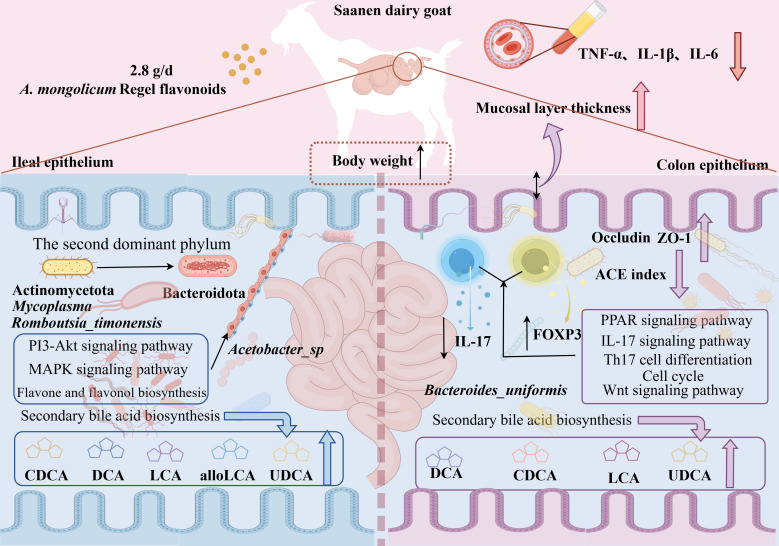
Effects of *A. mongolicum* Regel flavonoids on bile acid metabolism and microbial community in the ileum and colon of dairy goats. Supplementation with *A. mongolicum* Regel flavonoids in the diet of goats led to an increase in body weight and a reduction in the serum levels of the inflammatory factors TNF-α, IL-1β, and IL-6. The levels of CDCA, DCA, LCA, alloLCA, and UDCA in the ileum were significantly increased, whereas the levels of CDCA, DCA, LCA, and UDCA in the colon were also significantly elevated. In the ileum, the second most dominant phylum shifted from Actinomycetota to Bacteroidota, accompanied by alterations in the core microbial community. The main metabolic pathways involved include PI3-Akt, MAPK, flavone and flavonol biosynthesis, and secondary bile acid biosynthesis. The colonic mucosal thickness and the abundance of tight junction proteins increased, while the levels of pro-inflammatory cytokines decreased. The ACE index of intestinal bacteria was reduced, and the core microbial community underwent alterations. The major metabolic pathways involved include PPAR, IL-17, Wnt, Th17 cell differentiation, and secondary bile acid biosynthesis.

The present study demonstrated that dietary supplementation with *A. mongolicum* Regel flavonoids was associated with reduced serum levels of inflammatory cytokines, increased colonic mucosal thickness, improved intestinal barrier function, and altered concentrations of multiple BAs (CDCA, DCA, LCA, UDCA, and alloLCA) in the intestinal contents of goats. Shifts in microbial community structure and BAs metabolism were correlated with improvements in intestinal immune parameters, suggesting that *A. mongolicum* Regel flavonoids may exert a modulatory influence on host intestinal immune function via the gut microbiota–BA axis (Fig. 8). Collectively, these findings provide novel insights into the interrelationships among plant-derived flavonoids, the gut microbiota, BAs metabolism, and intestinal immunity.

## Supplementary Material

Reviewer comments

## Data Availability

The metagenomic data are available in the NCBI database under accession PRJNA1298777.

## SUPPLEMENTAL MATERIAL

Supplemental Figures msystems.00775-26-s0001.docx Figures S1 through S4.

Supplemental Tables msystems.00775-26-s0002.docx Tables S1 through S5.

OPEN PEER REVIEW reviewer-comments.pdf An accounting of the reviewer comments and feedback.

ASM does not own the copyrights to Supplemental Material that may be linked to, or accessed through, an article. The authors have granted ASM a non-exclusive, world-wide license to publish the Supplemental Material files. Please contact the corresponding author directly for reuse.
