# Supplementary material for: Allium mongolicum Regel flavonoids promote intestinal health in goats by regulating bile acid metabolism and gut microbiota
Source: mSystems. 2026 Jul 29;11(8):e00775-26. doi: 10.1128/msystems.00775-26 (PMC13483252; doi:10.1128/msystems.00775-26)
Supplement: Supplementary file 2 [file msystems.00775-26-s0001.docx]

**Fig. S1** Immunofluorescence staining of T_H_17 and T_reg_ cells in colon tissue. (A) T_H_17 cells (IL-17), T_reg_ cells (FOXP3), and nuclear staining (DAPI) for the CON and AMRF groups. (B) Statistical analysis of IL-17- and FOXP3-positive tissue percentages.

Abbreviation: CON: Basal diet group, AMRF: *Allium mongolicum* Regel flavonoids group. Significance is indicated as **P* < 0.05 and ***P* <0.01.

**Fig. S2** Unsupervised principal component analysis (PCA), supervised orthogonal partial least squares discriminant analysis (OPLS-DA), and displacement tests of the identified metabolites. (A) ileum, (B) colon.

**Fig. S3** Distribution of the top 20 microorganisms in terms of relative abundance at the phylum, genus, and species levels in the ileum and colon. Top 20 bacteria with relative abundance at the phylum (A), genus (B), and species (C) level in the ileum. Top 20 bacteria with relative abundance at the phylum (D), genus (E), and species (F) level in the colon.

Abbreviation: CON: Basal diet group, AMRF: *Allium mongolicum* Regel flavonoids group.

**Fig. S4** DESeq2‑based validation of differential bacteria. Bar chart of differential bacteria in the ileum (A). Bar chart of differential bacteria in the colon (B). Red indicates significant upregulation, and green indicates significant downregulation in the CON vs. AMRF comparison.
