# Supplementary material for: Allium mongolicum Regel flavonoids promote intestinal health in goats by regulating bile acid metabolism and gut microbiota
Source: mSystems. 2026 Jul 29;11(8):e00775-26. doi: 10.1128/msystems.00775-26 (PMC13483252; doi:10.1128/msystems.00775-26)
Supplement: Supplementary file 3 [file msystems.00775-26-s0002.docx]

**Table S1.** Yield of flavonoids from dried *Allium mongolicum* Regel leaves and a comparison of the relative contents of the main bioactive compounds (DM basis)

| Main active compound | Molecular mass | Flavonoids from dried *Allium mongolicum* Regel leaf |
| --- | --- | --- |
| Flavone |  |  |
| Crysoeriol O-glucuronic acid | 476.10 | 26900 |
| Apigenin | 270.05 | 665000 |
| Chrysoeriol 7-O-hexoside | 462.20 | 108000 |
| Tricin 7-O-hexoside | 492.10 | 319000 |
| Chrysoeriol | 300.06 | 639000 |
| Velutin | 314.08 | 48200 |
| Flavonol |  |  |
| Isorhamnetin | 316.06 | 4570000 |
| Rhamnetin (7-O-methxyl quercetin) | 316.06 | 72500 |
| Laricitrin | 332.05 | 27800 |
| Quercetin | 302.04 | 4760000 |
| Kumatakenin | 314.08 | 63100 |
| Morin | 302.04 | 40700000 |
| Flavanone |  |  |
| Homoeriodictyol | 302.28 | 2510000 |
| Isosakuranetin (4'-Methylnaringenin) | 286.08 | 102000 |
| Hesperetin | 302.08 | 62400 |
| Flavone C-glycosides |  |  |
| Luteolin 6-C-glucoside | 448.10 | 189000 |
| Chrysoeriol 8-C-hexoside | 462.10 | 27800 |
| Isoflavone |  |  |
| Biochanin A | 284.07 | 62400 |
| Prunetin | 284.07 | 78000 |
| Terpenoids |  |  |
| Phytocassane C | 318.10 | 108000 |
| Organic acids |  |  |
| 2-Hydroxybutanoic acid | 104.05 | 602000 |
| α-Hydroxyisobutyric acid | 104.05 | 147000 |
| (R)-lipoic acid | 206.04 | 4330 |
| Rosmarinic acid | 360.30 | 4930 |
| 2-Hydroxyisocaproic acid | 132.08 | 1520000 |
| D-Pantothenic dcid | 476.17 | 11000 |
| Phenolamides |  |  |
| N-p-Coumaroyl hydroxyagmatine | 292.20 | 31900 |
| Hydroxycinnamoyl derivatives |  |  |
| Caffeic aldehyde | 164.10 | 182000 |
| Syringaldehyde | 182.10 | 34400 |
| Alkaloids |  |  |
| Isoquinoline | 129.10 | 12800 |
| Phytohormones |  |  |
| Gibberellin A20 | 332.16 | 1160 |
| N6-Isopentenyladenine | 203.12 | 6760 |
| Methyl indole-3-acetate | 189.08 | 10800 |
| Indole 3-acetic acid | 175.06 | 134000 |
| Anthocyanins |  |  |
| Cyanidin 3-O-rutinoside | 595.00 | 60700 |
| Alcohols and polyols |  |  |
| DL-threitol | 122.06 | 134000 |

**Table S2** Effect of *A. mongolicum* *Regel* flavonoids on the ileal bile acid content in dairy goats

| Item, ng/g | Treatments | | SEM | FDR |
| --- | --- | --- | --- | --- |
|  | CON | AMRF |  |  |
| TβMCA | 8020.80 | 11039.67 | 493.48 | <0.001 |
| TCDCA | 6736.52 | 9641.65 | 470.01 | <0.001 |
| TUDCA | 5791.57 | 8228.77 | 398.37 | <0.001 |
| THCA | 4026.33 | 5350.98 | 223.11 | <0.001 |
| TCA | 2909.37 | 3954.00 | 169.95 | <0.001 |
| DCA | 1481.28 | 2408.21 | 143.40 | <0.001 |
| CA | 1046.59 | 1774.80 | 111.81 | <0.001 |
| NorCA | 2409.42 | 2882.36 | 114.94 | 0.059 |
| UDCA | 104.15 | 205.79 | 15.79 | <0.001 |
| βHDCA | 45.76 | 129.70 | 13.06 | <0.001 |
| CDCA | 57.27 | 130.22 | 11.49 | <0.001 |
| TLCA | 177.96 | 256.69 | 15.03 | 0.005 |
| UCA | 247.01 | 323.91 | 14.99 | 0.007 |
| GDCA | 67.36 | 121.52 | 8.40 | <0.001 |
| GCA | 182.98 | 229.59 | 8.39 | 0.002 |
| THDCA | 43.62 | 85.69 | 6.88 | <0.001 |
| βUDCA | 279.88 | 327.83 | 10.09 | 0.017 |
| LCA | 19.29 | 43.14 | 3.99 | <0.001 |
| 3-DHCA | 36.04 | 56.84 | 3.61 | <0.001 |
| HDCA | 40.56 | 21.31 | 3.66 | 0.005 |
| alloLCA | 26.23 | 39.81 | 2.71 | 0.009 |
| TDCA | 49.56 | 38.67 | 2.22 | 0.012 |
| ACA | 5.56 | 8.76 | 0.58 | 0.002 |
| PBAs | 22978.45 | 32132.08 | 1467.53 | <0.001 |
| SBAs | 10847.84 | 15139.11 | 689.06 | <0.001 |
| PBAs/SBAs | 2.12 | 2.12 | 0.02 | 0.982 |
| TBAs | 33826.28 | 47271.18 | 2149.16 | <0.001 |
| CBAs | 28048.13 | 38905.16 | 1753.08 | <0.001 |
| FBAs | 5779.78 | 8371.91 | 411.24 | <0.001 |
| CBAs/FBAs | 4.85 | 4.66 | 0.07 | 0.394 |

Abbreviation: CON: Basal diet group, AMRF: *Allium mongolicum* Regel flavonoids group.

**Table S3.** Effect of *A. mongolicum Regel* flavonoids on the colonic bile acid content in dairy goats

| Item, ng/g | Treatments | | SEM | FDR |
| --- | --- | --- | --- | --- |
|  | CON | AMRF |  |  |
| NorCA | 2831.02 | 6927.05 | 633.75 | <0.001 |
| βUDCA | 13297.91 | 17088.28 | 664.10 | <0.001 |
| DCA | 12495.00 | 14918.66 | 448.87 | 0.003 |
| βHDCA | 3295.53 | 4671.29 | 222.61 | <0.001 |
| CDCA | 3347.26 | 4586.78 | 204.41 | <0.001 |
| 6-ketoLCA | 628.85 | 1109.07 | 75.28 | <0.001 |
| HDCA | 1983.90 | 2403.12 | 77.52 | 0.003 |
| apoCA | 262.18 | 584.77 | 64.42 | 0.009 |
| TCDCA | 1177.73 | 1431.18 | 49.26 | 0.007 |
| TUDCA | 1152.68 | 1370.18 | 40.98 | 0.004 |
| TCA | 410.64 | 426.38 | 8.07 | 0.543 |
| TβMCA | 866.37 | 892.84 | 18.70 | 0.749 |
| THCA | 399.64 | 383.23 | 7.71 | 0.492 |
| UCA | 385.25 | 217.93 | 27.92 | <0.001 |
| 12-ketoLCA | 199.00 | 338.72 | 22.22 | <0.001 |
| 3-DHCA | 304.01 | 183.71 | 19.07 | <0.001 |
| CA | 324.02 | 432.95 | 18.15 | <0.001 |
| UDCA | 50.53 | 134.50 | 12.97 | <0.001 |
| isoLCA | 107.72 | 46.66 | 9.51 | <0.001 |
| 7-ketoLCA | 33.84 | 80.98 | 7.22 | <0.001 |
| LCA | 160.78 | 191.20 | 6.80 | 0.032 |
| GCA | 15.45 | 36.74 | 3.34 | <0.001 |
| GDCA | 20.26 | 37.57 | 2.98 | <0.001 |
| THDCA | 25.37 | 40.44 | 2.46 | <0.001 |
| alloLCA | 192.00 | 209.57 | 4.27 | 0.058 |
| TDCA | 8.03 | 5.50 | 0.46 | 0.002 |
| TLCA | 34.75 | 39.19 | 1.31 | 0.160 |
| 12-DHCA | 36.70 | 35.22 | 1.14 | 0.775 |
| dehydroLCA | 136.89 | 118.71 | 5.75 | 0.201 |
| ACA | 54.40 | 47.34 | 2.31 | 0.217 |
| NorDCA | 10.06 | 10.33 | 0.32 | 0.969 |
| PBAs | 6595.48 | 8237.43 | 282.98 | <0.001 |
| SBAs | 37662.23 | 50762.62 | 2141.55 | <0.001 |
| PBAs/SBAs | 0.18 | 0.16 | 0.00 | <0.001 |
| TBAs | 44257.72 | 59000.05 | 2420.63 | <0.001 |
| CBAs | 4110.90 | 4663.23 | 114.37 | 0.007 |
| FBAs | 40146.82 | 54336.82 | 2315.87 | <0.001 |
| CBAs/FBAs | 0.10 | 0.09 | 0.00 | <0.001 |

Abbreviation: CON: Basal diet group, AMRF: *Allium mongolicum* Regel flavonoids group.

**Table S4** Results of KEGG pathway enrichment analysis of microbial communities in the ileum

| Items | *P* value | Fold Change | FDR | Lower ci | Upper ci | Effect size |
| --- | --- | --- | --- | --- | --- | --- |
| Fatty acid biosynthesis | 0.04533 | 1.6224 | 0.4027 | 0.02543 | 0.2459 | 0.1316 |
| Arginine and proline metabolism | 0.04533 | 1.34281 | 0.4027 | 0.0188 | 0.1261 | 0.07336 |
| PI3K-Akt signaling pathway | 0.06508 | 1.25006 | 0.4027 | -0.02342 | 0.0343 | 0.00916 |
| Th17 cell differentiation | 0.1727 | 1.10347 | 0.4751 | -0.02955 | 0.02887 | 0.00354 |
| Flavone and flavonol biosynthesis | 0.0078 | 4.21481 | 0.1865 | 0.00461 | 0.01886 | 0.01104 |
| FoxO signaling pathway | 0.00477 | 7.88647 | 0.1818 | 0.00662 | 0.02189 | 0.01356 |
| Cholesterol metabolism | 0.0078 | 3.94673 | 0.1865 | 0.00441 | 0.0101 | 0.0074 |
| Calcium signaling pathway | 0.00477 | 4.38428 | 0.1818 | 0.00106 | 0.00575 | 0.00304 |
| Secondary bile acid biosynthesis | 0.1727 | 1.6475 | 0.4751 | -0.00256 | 0.04041 | 0.01992 |
| MAPK signaling pathway | 0.07401 | 0 | 0.4028 | 5.00E-05 | 0.0026 | 0.00124 |

**Table S5** Results of KEGG pathway enrichment analysis of microbial communities in the colon

| Items | *P* value | Fold Change | FDR | Lower ci | Upper ci | Effect size |
| --- | --- | --- | --- | --- | --- | --- |
| PPAR signaling pathway | 0.3785 | 1.01868 | 0.7847 | -0.00276 | 0.00764 | 0.00294 |
| Secondary bile acid biosynthesis | 0.5752 | 0.99686 | 0.8441 | -0.0048 | 0.00455 | -0.0002 |
| IL-17 signaling pathway | 0.1282 | 0.9396 | 0.6585 | -0.00591 | 8.00E-05 | -0.00299 |
| Th17 cell differentiation | 0.1282 | 0.93948 | 0.6585 | -0.00578 | 0.0002 | -0.00299 |
| Carbohydrate digestion and absorption | 0.04533 | 1.14994 | 0.6585 | 0.0017 | 0.00906 | 0.00531 |
| Metabolism of xenobiotics by cytochrome P450 | 0.8102 | 0.98851 | 0.9463 | -0.00218 | 0.00151 | -0.00032 |
| Flavone and flavonol biosynthesis | 0.02024 | 0.81469 | 0.6585 | -0.00558 | -0.00133 | -0.00331 |
| Bile secretion | 0.0927 | 0.65955 | 0.6585 | -0.00202 | -4.00E-05 | -0.00106 |
| Cell cycle | 0.04533 | 1.8811 | 0.6585 | 0.00012 | 0.00094 | 0.00049 |
| Wnt signaling pathway | 0.06555 | 1.90466 | 0.6585 | -2.49E-06 | 0.00049 | 0.00024 |
